# Induction of SARS-CoV-2-Specific IgG and IgA in Serum and Milk with Different SARS-CoV-2 Vaccines in Breastfeeding Women: A Cross-Sectional Study in Northern Spain

**DOI:** 10.3390/ijerph18168831

**Published:** 2021-08-21

**Authors:** Carolina Lechosa-Muñiz, María Paz-Zulueta, Jose Manuel Mendez-Legaza, Juan Irure-Ventura, Rocío Cuesta González, Jorge Calvo Montes, Marcos López-Hoyos, Javier Llorca, María Jesús Cabero-Pérez

**Affiliations:** 1 Faculty of Nursing, University of Cantabria, Avda Valdecilla s/n. C.P., 39008 Santander, Cantabria, Spain; carolina.lechosa@unican.es; 2 Breastfeeding Coordinator, IBCLC, Hospital Universitario Marqués de Valdecilla, C.P., 39008 Santander, Cantabria, Spain; 3 Grupo de Investigación en Derecho Sanitario y Bioética, GRIDES, IDIVAL, C/ Cardenal Herrera Oria s/n. C.P., 39011 Santander, Cantabria, Spain; 4 Department of Microbiology, Hospital Universitario Marqués de Valdecilla, C.P., 39008 Santander, Cantabria, Spain; josemanuel.mendez@scsalud.es (J.M.M.-L.); jorge.calvo@scsalud.es (J.C.M.); 5 Department of Immunology, Hospital Universitario Marqués de Valdecilla, IDIVAL, C.P., 39008 Santander, Cantabria, Spain; juan.irure@scsalud.es (J.I.-V.); marcos.lopez@scsalud.es (M.L.-H.); 6 Department of Pediatrics, Hospital Universitario Marqués de Valdecilla, C.P., 39008 Santander, Cantabria, Spain; rociocuestagonzalez@gmail.com; 7 Laboratory, Molecular Biology Department, University of Cantabria, Avenida del Cardenal Herrera Oria 2, C.P., 39010 Santander, Cantabria, Spain; 8 Faculty of Medicine, University of Cantabria, Avenida del Cardenal Herrera Oria 2, C.P., 39010 Santander, Cantabria, Spain; javier.llorca@unican.es (J.L.); mariajesus.cabero@unican.es (M.J.C.-P.); 9 CIBER Epidemiology and Public Health (CIBERESP), C.P., 28029 Madrid, Spain; 10 Pediatrics Section, Hospital Universitario Marqués de Valdecilla, C.P., 39008 Santander, Cantabria, Spain; 11 IDIVAL, C/ Cardenal Herrera Oria s/n. C.P., 39011 Santander, Cantabria, Spain

**Keywords:** antibodies, breastfeeding, breast milk, COVID-19 vaccine, maternal immunity, neonatal immunity

## Abstract

Breastfeeding mothers were excluded from the clinical trials conducted for vaccines against SARS-CoV-2. Since the start of the vaccination, some doubts have arisen regarding its compatibility with breastfeeding. The aim of this study was to analyse the presence of anti-SARS-CoV-2 antibodies in breast milk and serum (IgG and IgA) of vaccinated breastfeeding women. The main variables of the observational study were: adverse related events after vaccination and determination of the presence of IgG and IgA isotypes antibodies in serum and in breast milk of vaccinated women against the SARS-CoV-2 antigens. Results: 110 breastfeeding mothers were included; 70 women (63.6%) were vaccinated with two doses of BNT162b2, 20 women (18.2%) with two doses of mRNA-1273, and 20 women (18.2%) with a single dose of ChAdOx1-S. Regarding adverse reactions and vaccine safety, 38 women had no adverse reactions; 20 (18.2%) had general malaise or adenopathies; 10 (9.1%) had a headache; and 7 (6.4%) had fever. When analysing IgG antibodies, significantly higher levels of antibodies were found in serum and breast milk from mothers vaccinated with BNT162b2 or mRNA-1273 vs. ChAdOx1-S (*p* < 0.001 and *p* = 0.001, respectively). Analysing IgA antibodies, significant differences were found when comparing mean values in serum from mothers vaccinated with BNT162b2 or mRNA-1273 vs. ChAdOx1-S (0.12, 0.16, and 0.02, respectively; *p* < 0.001) and breast milk of mothers vaccinated when comparing BNT16b2 vs. ChAdOx1-S. All vaccinated breastfeeding mothers had serum anti-S1 IgG antibodies in response to vaccination against SARS-CoV-2, regardless of the commercial vaccine administered. Conclusions: the anti-SARS-CoV-2 vaccines were well tolerated by the mothers and the breastfed infant. In addition, breastfeeding mothers offer their infants IgA and IgG isotype antibodies directed against SARS-CoV-2 protein S in breast milk.

## 1. Introduction

The World Health Organization and scientific societies in America and Europe recommend exclusive breastfeeding for up to 6 months and then continued breastfeeding combined with solid foods for 2 years or more for as long as the mother and the baby desire [1,2,3]. Breastfeeding is one of the most efficacious tools for preventing diseases and for promoting health in both mothers and children [4]. Among its short-term beneficial effects, breastfeeding was shown to reduce the incidence and/or the severity of infectious, gastrointestinal, respiratory, acute otitis media and urinary tract infections in infants, mainly through the transfer of maternal antibodies [5,6,7,8,9].

In addition, the World Health Organization recommends exclusive breastfeeding for the first 6 months of life, even if the mother is infected with the new SARS-CoV-2 coronavirus [10]. Breast milk from women infected with SARS-CoV-2 was tested for SARS-CoV-2 specific antibodies in breast milk [11]. Although RNA particles of the virus were detected in about 10% of breast milk samples tested [12], neither viable nor transmissible SARS-CoV-2 was detected in breast milk, and there are no documented cases of transmission to the infant through breast milk [13,14].

The SARS-CoV-2 vaccination process started at the end of December 2020 in Spain. Since the start of the vaccination, some doubts have arisen regarding its compatibility with breastfeeding. The reason for this is the lack of evidence due to the fact that breastfeeding mothers were not included in clinical trials prior to the commercialisation of the vaccines. Laboratories warn in their data sheets about the lack of evidence of secretion in breast milk [15]. In contrast, breastfeeding mothers belonging to priority groups such as health care workers or workers in nursing homes were offered to be vaccinated under the consideration that the proved benefits of the vaccination would overcome any presumptive side effect.

At present, there is no justification for withholding breastfeeding as a condition for administering the SARS-CoV-2 vaccines [16,17,18]. On the other hand, a possible mechanism of protection could be passive immunity through breastfeeding from a previously vaccinated mother. The extent to which breastfeeding of newborns may have specific immunological protection against SARS-CoV-2 is still unknown, although breast milk was shown to be protective against infection due to its specific protection attributable to secretory immunoglobulin IgA as well as containing numerous bioactive factors [19,20,21].

The main objectives of our study were to detect the presence of IgG and IgA antibodies directed against SARS-CoV-2 protein S in blood from breastfeeding women and to detect the presence of IgA and IgG isotype antibodies directed against SARS-CoV-2 protein S in breast milk.

## 2. Materials and Methods

A cross-sectional study was performed, including 110 breastfeeding non-infected mothers recruited at University Hospital Marques de Valdecilla, Santander, Spain. The study period was from 1 April 2021 to 30 April 2021. Another 23 breastfeeding mothers who were non-infected and non-vaccinated were included as a control group.

The study was disseminated to the community of healthcare professionals through their corporate work e-mails. It was also disseminated through social networks informing about the study and how to contact the principal investigator. Women interested in participating in the study received informed consent. Participants then underwent a structured interview for data collection and sample collection. All lactating women who received both doses of the vaccine were included in the study, except for women vaccinated with ChAdOx1-S, who were included with one dose. It is of note that vaccination with ChAdOx1-S was halted during the study because of the appearance of severe episodes of vaccine-induced immune thrombotic thrombocytopenia; therefore, the second dose of breastfeeding mothers who received the first dose of ChAdOx1-S was delayed. Thus, women recruited in our study had received two doses of either mRNA-based vaccines (BNT162b2 or mRNA-1273) or just one dose of ChAdOx1-S vaccine.

Clinical data were obtained at the moment of samples collection through direct interview. The reported data during interview comprised age of the breastfeeding mothers, maternal educational level, employment, parity, personal health history, pregnancy pathology, current treatment, breastfeeding type (exclusive or mixed with artificial formula), infant age, vaccine type (BNT162b2, mRNA-1273, or ChAdOx1-S) and lot, vaccination dates, adverse effects on mother (none, local pain, fever, general malaise, adenopathy, headache, nausea, and other) and infant after each dose, and history of SARS-CoV-2 infection.

### 2.1. Sample Collection and Processing

Blood and breast milk from breastfeeding women who received BNT162b2 or mRNA-1273 SARS-CoV-2 vaccines were collected 30 days after the second dose of the vaccine (mean 30.3; SD 0.56). Samples from women vaccinated using ChAdOx1-S were collected 30 days after the first dose (mean 30.4; SD 0.60). Sampling consisted of 1 mL of milk and 5 mL of venous blood without anticoagulants. Blood was centrifuged at 3000 revolutions per minute (rpm) for 10 min at room temperature, and sera were aliquoted in cryogenic vials and stored at −20 °C until use. Breast milk was collected into breast milk bottles, centrifuged at 2000 rpm at 4 °C for 25 min, and the supernatant was aliquoted into cryogenic vials and stored at −20 °C until use. Prior to processing, breast milk samples were thawed, centrifuged at 2000 rpm for 15 min, fat was removed, and supernatant was transferred to a new tube. Centrifugation was repeated 2× to ensure removal of all cells and fat.

All serum and breast milk samples were tested in parallel on two different SARS-CoV-2 antibodies testing platforms, which are described in detail below.

### 2.2. IgG and IgA Antibody Detection by ELISA

The detection of IgG and IgA antibodies in serum samples and in breast milk against SARS-CoV-2 was performed by ELISA following IrsiCaixa published protocol [22].

Serum samples were previously diluted 1:100 in phosphate buffer saline (PBS), and breast milk samples were used without dilution.

Briefly, Nunc MaxiSorp 96-well plates (Thermo Fisher Scientific, Waltham, MA, USA) were coated with optimized concentrations of 2 ug/mL of capture antibody (MA1-21315, Thermo Fisher Scientific) diluted with phosphate buffer saline (PBS) overnight at 4 °C. Coated plates were washed and blocked with PBS 1× + 1% bovine serum albumin (BSA) for two hours at room temperature. After washing the plates, antigen solution (S2 + RBD (Sino Biological, Beijing, China) diluted in blocking buffer) was added to one half of the plate, and blocking buffer was added to the other half and incubated overnight at 4 °C. Serum and breast milk samples were added and incubated for one hour at room temperature. Then, incubation with peroxidase-conjugated anti-IgG and anti-IgA detection antibodies (Jackson Immunoresearch, West Grove, PA, USA) was carried out for 30 min at room temperature. Bound antigen-specific antibodies were detected by adding the substrate solution. Absorbance was read at 492 nm. The specific signal associated with each sample was calculated by background subtraction as follows: AU specific signal = OD (+Ag) − OD (−Ag). Results with an optical density (AU) >0.1 after the background subtraction were considered positive.

### 2.3. Anti-S1 IgG Antibody Detection by Chemiluminescent Microparticle Immunoassay (CLIA)

Serum and br3east milk samples from lactating mothers were additionally evaluated for the detection of anti-SARS-CoV-2 S1 (RBD) protein-targeting IgG antibodies. Serum as well as breast milk samples were both tested by chemiluminescent microparticle immunoassay (CLIA) using the SARS-CoV-2 IgG II Quant Assay on the Alinity (Abbott, Abbott Park, IL, USA). This CLIA assay is used for the detection of anti-SARS-CoV-2 S1 (RBD) protein-targeting antibodies in human serum and plasma and is currently not validated for other samples. Milks from lactating mothers were also analysed with this assay after the centrifugation process mentioned above. For the interpretation of the value as a positive result in the determination of antibodies, it was considered positive above a value of 50 AU/mL in serum samples, following the manufacturer’s indications. In the case of breast milk samples, since the use of the technique has not been validated for this type of sample, no cutoff point was established. However, in order to reduce or avoid possible analytical interferences in the determination of antibodies in breast milk, since it is a more heterogeneous sample, the arithmetic mean of the values obtained in the milks of the control group (unvaccinated lactating mothers) was subtracted from the result of each breast milk sample.

## 3. Statistical Analysis

An initial descriptive analysis was incorporated. For the categorical and the discrete variables, proportions were estimated by their corresponding 95% confidence intervals (95% CI) using Pearson’s chi-squared test for comparisons, or, alternatively, using Fisher’s exact test when over 20% of the fields presented a number of expected cases that was fewer than or equal to five. For the continuous variables, mean, standard deviation (SD), median, and interquartile range were estimated. Mean values of antibodies according to the three types of vaccine were compared using ANOVA. If the result was significant (ANOVA *p* value < 0.05), we carried out a pairwise post hoc analysis using the Student’s t test. In this case, *p* values were corrected for multiple comparisons using the Sidak’s method. Correlation between different types of antibodies was studied using Pearson’s linear correlation coefficient. The alpha error was set at 0.05, and all *p* values were bilateral. Statistical analysis was performed using the SPSS v22.0 (IBM SPSS Statistics for Windows. IBM Corp.: Armonk, NY, USA) and Stata 16/SE (StataCorp, College Station, TX, USA).

## 4. Ethics Approval and Consent to Participate

The study was approved by the Clinical Research Ethics Committee of Cantabria (12 March 2021) (project identification code 2021.073). All subjects gave their informed consent for inclusion before they participated in the study. The study was conducted in accordance with the Declaration of Helsinki, modification of Fortaleza (2013). The data were pseudo-anonymized and processed in a confidential way according to the regulation (UE) 2016/679 (27 April 2016) on the protection of natural persons with regard to personal data processing and free movement of this data and the Spanish organic law 3/2018 (5 December) about personal data protection and guarantee of digital rights. Each patient was identified with a unique specific code making compatible the confidentiality and the follow-up of medical data. Likewise, specific security measures were taken to prevent the re-identification and the access of unauthorized third parties.

## 5. Results

In total, 110 breastfeeding mothers were included. The mean age of women was 37.1 ± 3.9, ranging from 27 to 46 years. Of these, 103 women (93.6%) had a university education, and 85 women (77.3%) worked in the health sector. About half of the women were primiparous (61, 55.5%). Regarding the health status of the women in our sample, 87 were women with no relevant personal history, hypothyroidism being the most frequent diagnosis (*n* = 9, 8.2%), 85 women had no pregnancy pathology, and 94 were not currently receiving any treatment. The remaining characteristics of the sample are detailed in Table 1.

In relation to the vaccines, 70 women (63.6%) were vaccinated with two doses of BNT162b2, 20 women (18.2%) with two doses of miRNA-1273, and 20 women (18.2%) with one dose of ChAdOx1-S (Table 1).

In relation to the infants, 59 (53.6%) were male, and 51 (46.4%) were female. Up to 94.5% were full-term births, and 5.5% were preterm births. The mean weight at birth was 3236 ± 481.2 g, ranging from 1570 to 4650 g. The mean age at inclusion in the study was 15.9 ± 11.2 months, ranging from 2.2 to 63.3 months. Of these, 94 (85.5%) infants were exclusively breastfed or were exclusively breastfed up to 6 months, and 86 (78.2%) of the infants studied did not attend nursery school (Table 2).

Regarding adverse reactions and vaccine safety, 38 of the women studied had no adverse reactions, 20 (18.2%) had general malaise or adenopathies, 10 (9.1%) had a headache, and 7 (6.4%) had fever (Table 3). Only one breastfed infant was more irritable for 24 h (data not shown in table). No mother or infant had a serious adverse reaction requiring either emergency care or admission at hospital.

When analysing IgG, the mean antibody titres observed in the serum of lactating mothers were different according to the type of vaccine they received, being 0.32, 0.30, and 0.16 (AU) for mothers who received BNT162b2, mRNA-1273, and ChAdOx1-S (one dose), respectively. The mean antibody titres observed in milk were also different according to the type of vaccine received, being 0.41, 0.45, and 0.09 (AU) for mothers who received BNT162b2, mRNA-1273, and ChAdOx1-S (one dose), respectively. Significant differences were found when comparing mean of IgG antibodies in both serum and milk of mothers vaccinated with BNT162b2 or mRNA-1273 vs. ChAdOx1-S (Sidak method), but no differences could be found between those vaccinated with BNT162b2 vs. mRNA-1273 (Table 4).

When analysing IgA, the mean antibody titres observed in the serum of lactating mothers showed differences according to the type of vaccine administered: 0.12, 0.16, and 0.02 (AU) for mothers who received BNT162b2, mRNA-1273, and ChAdOx1-S (one dose) vaccines, respectively. The mean antibody titres observed in milk were also different according to the type of vaccine administered, being 0.11, 0.10, and 0.04 (AU) for mothers who received BNT162b2, mRNA-1273, and ChAdOx1-S (one dose), respectively. Significant differences were found when comparing means serum of mothers vaccinated with BNT162b2 or mRNA-1273 vs. ChAdOx1-S and the milk of mothers vaccinated with comparing BNT16b2 vs. ChAdOx1-S (Sidak method; Table 4 and Supplementary Figure S1).

A significant correlation was observed between serum IgG and IgA levels in mothers who received BNT162b2 and mRNA-1273 vaccines (*p* < 0.001). No significant correlation was observed between these same parameters for mothers who received ChAdOx1-S vaccine or for the rest of the parameters analysed in the study (Table 5 and Supplementary Figure S2).

All vaccinated breastfeeding mothers had serum anti-S1 IgG antibodies in response to vaccination against SARS-CoV-2, regardless of the commercial vaccine administered. On the other hand, the presence of anti-S1 IgG antibodies was not observed in unvaccinated mothers as expected.

Statistically significant differences in anti-S1 IgG antibodies were observed in serum but not in breast milk of lactating mothers depending on the type of vaccine received (ANOVA with Sidak correction for multiplicity) (Table 6). The mean serum IgG anti-S1 antibody titre was significantly higher in mothers vaccinated with vaccine BNT162b2 and mRNA-1273 than in mothers vaccinated with ChAdOx1-s (ANOVA; *p* < 0.001).

Mean serum IgG anti-S1 antibody titres were 8968, 11642, and 467 AU/mL for mothers who received BNT162b2, mRNA-1273, and ChAdOx1-S vaccine, respectively. Mean titres of IgG anti-S1 antibodies in the milk samples were also different according to the type of vaccine received, being 90.0, 60.1, and 0.9 AU/mL for those mothers who received BNT162b2, mRNA-1273, and ChAdOx1-S vaccines, respectively.

## 6. Discussion

Breastfeeding mothers were excluded from all pre-marketing trials of anti-SARS-CoV-2 vaccines. Our study shows that the anti-SARS-CoV-2 vaccines used are safe for both the mother and the breastfed infant. No serious adverse effects occurred in either mother or infant during our study period. Our results are consistent with those recently reported for breastfeeding vaccinated women [23,24] and showed no differences from those reported for non-breastfeeding vaccinated women [25].

In addition to safety, immunity was shown to have similar results in obtaining IgG and IgA antibodies directed against the S protein of SARS-CoV-2 in serum and breast milk [26,27]. As an added value, breastfeeding mothers offer their infants IgA and IgG isotype antibodies directed against SARS-CoV-2 protein S in breast milk. Our results are consistent with those obtained in the study by Juncker et al. to determine the effect of vaccination on the levels of SARS-CoV-2 specific IgA in breast milk (*n* = 26). After the second dose with BNT162b2, they observed an accelerated IgA antibody and SARS-CoV-2 specific antibody response in breast milk [23]. Perl et al. (*n* = 84) showed that the mean levels of anti-SARS-CoV-2-specific IgA antibodies in the breast milk increased rapidly and were significantly elevated at 2 weeks after the first vaccine (*p* < 0.001), increasing 1 week after the second vaccine (BNT162b2). The mean levels of anti-SARS-CoV-2-specific IgG antibodies remained low for the first 3 weeks, with an increase at week four (*p* = 0.004) [24]. In addition, Collier et al. (*n* = 16 lactating women) evaluated the immunogenicity. This study validated that vaccination with BNT162b2 or mRNA-1273 elicits higher antibody responses than does infection [25].

As is the case after vaccination against other viruses, antibodies (mainly IgA and IgG) generated by the vaccine are excreted in the milk of lactating mothers vaccinated against SARS-CoV-2, which makes it possible that vaccinating lactating mothers could passively protect their children [25,26,27]. The clinical relevance of the levels of antibodies in breastmilk we reported, however, needs wider studies to be elucidated.

As the COVID-19 pandemic goes on, new cases in European countries are being progressively concentrated in younger people. A relevant public health consequence is the increasing risk for women in fertile age of getting infected by SARS-CoV-2, eventually leading to higher risk for both pregnant and lactating women and their children. Further advances in vaccination coverage in these ages, which have been mostly left aside thus far, are needed to safeguard them. In this sense, our results provide some base to extend anti-SARS-CoV-2 vaccination in lactating mothers.

The most important limitation of our study is the fact that women vaccinated using ChAdOx1-S received just one dose; thus, their antibody levels were not fully comparable with those who were fully vaccinated with BNT162b2 or mRNA-1273. There are several reasons for studying women with just a ChAdOx1-S dose. Firstly, the interval between doses of this vaccine is longer than for mRNA-based vaccines. Secondly, this study coincided with the alarming news regarding the putative association between ChAdOx1-S and severe venous thrombo-embolisms, which led to an initial delay in the administration of the second dose. Later, people with incomplete schedule of ChAdOx1-S were offered to complete their vaccination with a second dose of any mRNA-based vaccine [28]. These circumstances made it impossible to measure antibody levels in women fully vaccinated with ChAdOx1-S. A second limitation was the lack of longitudinal data, specifically the lack of pre-vaccination data. We cannot rule out that some women could have been infected with SARS-CoV-2 before being vaccinated. If that was the case, their antibody levels in our study would have not been a proper representation of their response to vaccination. However, women were allocated to vaccination as vaccines became available; therefore, previously infected women could have been assigned to any available vaccine in a blind manner, which makes a bias less likely. Furthermore, we had no data in the follow-up; therefore, we could not evaluate the persistence of antibodies over time.

Regarding the strengths of this study, we were able to study 110 lactating mothers vaccinated with two doses of BNT162b2 or miRNA-1273 or one dose of ChAdOx1-S. Literature about the immune response after vaccination against SARS-CoV-2 is still limited in lactating women. Therefore, in terms of implications for clinical practice, our results support the available evidence between safety vaccines and the presence of anti-SARS-CoV-2 antibodies in breast milk and serum (IgG and IgA) of vaccinated breast-feeding women.

## 7. Conclusions

Our study shows that the anti-SARS-CoV-2 vaccines used were well tolerated by mothers and breastfed infants. The anti-SARS-CoV-2 vaccine should not prevent the initiation of breastfeeding and does not require the interruption of breastfeeding.

In our study, we showed a positive correlation between antibody levels in serum and breast milk samples. However, those women who received ChAdOx1-S vaccine elicited lower levels of antibodies in serum and in breast milk samples, probably because they received only one dose at the moment of analysis. We would like to highlight the need of a second dose of the ChAdOx1-S vaccine.

As an added value, breastfeeding mothers offer their infants IgA and IgG isotype antibodies directed against SARS-CoV-2 protein S in breast milk.

## Figures and Tables

**Table 1:** Descriptive analysis of the breastfeeding mothers included during the study period (2021).

	All Participants *n* = 110	%	
Maternal age: mean (SD)	37.1	(3.9)	range: 27–46
Vaccine			
BNT162b2	70	63.6	
miRNA-1273	20	18.2	
ChAdOx1-S	20	18.2	
Maternal educational level			
Foundation degree	7	6.4	
University studies	103	93.6	
Employment			
Technicians/PCSE	22	20.0	
Technicians/support professionals	1	0.9	
Office with public	1	0.9	
Health and care	85	77.3	
Safety	1	0.9	
Parity			
Primiparous	61	55.5	
Multiparous	49	44.5	
Personal health history			
None	87	79.1	
Hypothyroidism	9	8.2	
Protein S deficiency	3	2.7	
Hyperthyroidism	2	1.8	
Asthma	2	1.8	
Celiac disease	2	1.8	
Other *	5	4.5	
Pregnancy pathology			
None	85	77.3	
Gestational diabetes (diet)	9	8.2	
Threatened miscarriage	3	2.7	
Small gestational age	3	2.7	
Intrauterine growth retardation I	2	1.8	
Other **	8	7.3	
Current treatment			
None	94	85.5	
Progesterone	9	8.2	
Levothyroxine	3	2.7	
Antidepressants	2	1.8	
Insulin	1	0.9	
Levothyroxine + antidepressants	1	0.9	

* other: polycystic ovary syndrome, thyroid cancer, epilepsy, hypothyroidism/asthma, or diabetes mellitus (type I) (frequency = 1). ** other: gestational diabetes (insulin), pre-eclampsia, NIPPD, premature rupture of membranes, hepatic cholestasis, gestational diabetes + placenta praevia, gestational diabetes + threatened miscarriage + cholestasis or pre-eclampsia + cholestasis + intrauterine growth retardation I (frequency = 1).

**Table 2:** Descriptive analysis of breastfed infants included during the study period (2021).

	All Participants *n* = 110	%	
Newborn gender			
Male	59	53.6	
Female	51	46.4	
Pregnancy duration			range: 31–41 + 6
≥37 weeks	104	94.5	
<37 weeks	6	5.5	
Newborn weight: mean (SD)	3236	(481.2)	range: 1570–4650
2500–4000 g	98	89.1	
>4000 g	6	5.4	
<2500	6	5.4	
Current age: mean (SD)	15.9	11.2	range: 2.2–63.3
0–6 months	14	12.7	
7–12 months	45	40.0	
13–24 months	32	29.1	
>24 months	19	17.3	
Type of feeding			
Exclusive breastfeeding	94	85.5	
No exclusive breastfeeding	16	14.5	
Number of milk feedings: mean (SD)	5.8	2.4	
Nursery attendance			
No	86	78.2	
Yes	24	21.8	

**Table 3:** Adverse related events after vaccination of breastfeeding mothers included during the study period (2021).

	BNT162b2	mRNA-1273	ChAdOx1-S	All Participants	
	*n*	*n*	*n*	*n*	%
None	30	2	6	38	34.5
General malaise	9	7	4	20	18.2
Adenopathy	10	3	7	20	18.2
Headache	4	4	2	10	9.1
Fever	5	2	0	7	6.4
Local pain	2	0	0	2	1.8
Nausea	1	0	0	1	0.9
Other *	9	2	1	12	10.9

* other minor adverse effects combined (frequency = 1).

**Table 4:** Levels of IgG and IgA in serum and breast milk according to the type of vaccine received.

Antibody	Vaccine	Mean ± Standard Deviation	ANOVA *p* Value	*p* *
IgG serum	BNT162b2	0.32 ± 0.13	<0.001	BNT162b2 vs. mRNA-1273: 0.97
	mRNA-1273	0.30 ± 0.10		BNT16b2 vs. ChAdOx1-S: <0.001
	ChAdOx1-S (one dose)	0.16 ± 0.09		mRNA-1273 vs. ChAdOx1-S: 0.001
IgA serum	BNT162b2	0.12 ± 0.10	<0.001	BNT162b2 vs. mRNA-1273: 0.38
	mRNA-1273	0.16 ± 0.13		BNT16b2 vs. ChAdOx1-S: <0.001
	ChAdOx1-S (one dose)	0.02 ± 0.02		mRNA-1273 vs. ChAdOx1-S: <0.001
IgG breast milk	BNT162b2	0.41 ± 0.10	<0.001	BNT162b2 vs. mRNA-1273: 0.18
	mRNA-1273	0.45 ± 0.08		BNT16b2 vs. ChAdOx1-S: <0.001
	ChAdOx1-S (one dose)	0.09 ± 0.08		mRNA-1273 vs. ChAdOx1-S: <0.001
IgA breast milk	BNT162b2	0.11 ± 0.12	0.01	BNT162b2 vs. mRNA-1273: 0.92
	mRNA-1273	0.10 ± 0.07		BNT16b2 vs. ChAdOx1-S: 0.02
	ChAdOx1-S (one dose)	0.04 ± 0.07		mRNA-1273 vs. ChAdOx1-S: 0.20

* *p* values comparing means, adjusted for multiple comparisons (Sidak method).

**Table 5:** Linear correlation coefficients between levels of IgG and IgA in serum and breast milk according to the type of vaccine received.

		Vaccine	r	*p*
IgG serum	IgA serum	BNT162b2	0.4812	<0.001
		mRNA-1273	0.5530	<0.008
		ChAdOx1-S (one dose)	0.2064	0.40
IgG serum	IgG breast milk	BNT162b2	0.1456	0.22
		mRNA-1273	0.0025	0.99
		ChAdOx1-S (one dose)	0.2151	0.38
IgG serum	IgA breast milk	BNT162b2	0.1088	0.36
		mRNA-1273	0.2234	0.32
		ChAdOx1-S (one dose)	−0.1100	0.65
IgA serum	IgG breast milk	BNT162b2	0.1548	0.19
		mRNA-1273	0.0183	0.94
		ChAdOx1-S (one dose)	−0.1510	0.54
IgA serum	IgA breast milk	BNT162b2	0.1142	0.34
		mRNA-1273	0.2429	0.28
		ChAdOx1-S (one dose)	−0.0831	0.74
IgG breast milk	IgA breast milk	BNT162b2	0.1569	0.18
		mRNA-1273	0.4662	0.03
		ChAdOx1-S (one dose)	0.2600	0.28

**Table 6:** Levels of anti-S1 antibodies (IgG) in serum and breast milk according to the type of vaccine received.

Antibody	Vaccine	Mean ± Standard Deviation	*p* *
Anti-S1 IgG serum (AU/mL)	BNT162b2	8968 ± 7394	BNT162b2 vs. mRNA-1273: 0.29
	mRNA-1273	11642 ± 7591	BNT16b2 vs. ChAdOx1-S: <0.001
	ChAdOx1-S (one dose)	467 ± 525	mRNA-1273 vs. ChAdOx1-S: <0.001
Anti-S1 IgG breast milk (AU/mL)	BNT162b2	90.0 ± 276.5	BNT162b2 vs. mRNA-1273: 0.93
	mRNA-1273	60.1 ± 60.6	BNT16b2 vs. ChAdOx1-S: 0.31
	ChAdOx1-S (one dose)	0.9 ± 1.2	mRNA-1273 vs. ChAdOx1-S: 0.78

* *p* values comparing means, adjusted for multiple comparisons (Sidak method).

## Data Availability

The data presented in this study are available on request from the corresponding author.

## Supplementary Materials

The following are available online at https://www.mdpi.com/article/10.3390/ijerph18168831/s1. Supplementary Figure S1. Levels of IgG and IgA in serum and breast milk according to the type of vaccine received. Supplementary Figure S2. Linear trend of the relationship between levels of IgG and IgA in serum and breast milk according to the type of vaccine received.
