# Supplementary material for: Induction of SARS-CoV-2-Specific IgG and IgA in Serum and Milk with Different SARS-CoV-2 Vaccines in Breastfeeding Women: A Cross-Sectional Study in Northern Spain
Source: Int J Environ Res Public Health. 2021 Aug 21;18(16):8831. doi: 10.3390/ijerph18168831 (PMC8393848; doi:10.3390/ijerph18168831)

Supplementary Figure S1. Levels of IgG and IgA in serum and breast milk, according to the type of vaccine received.

A) IgG serum

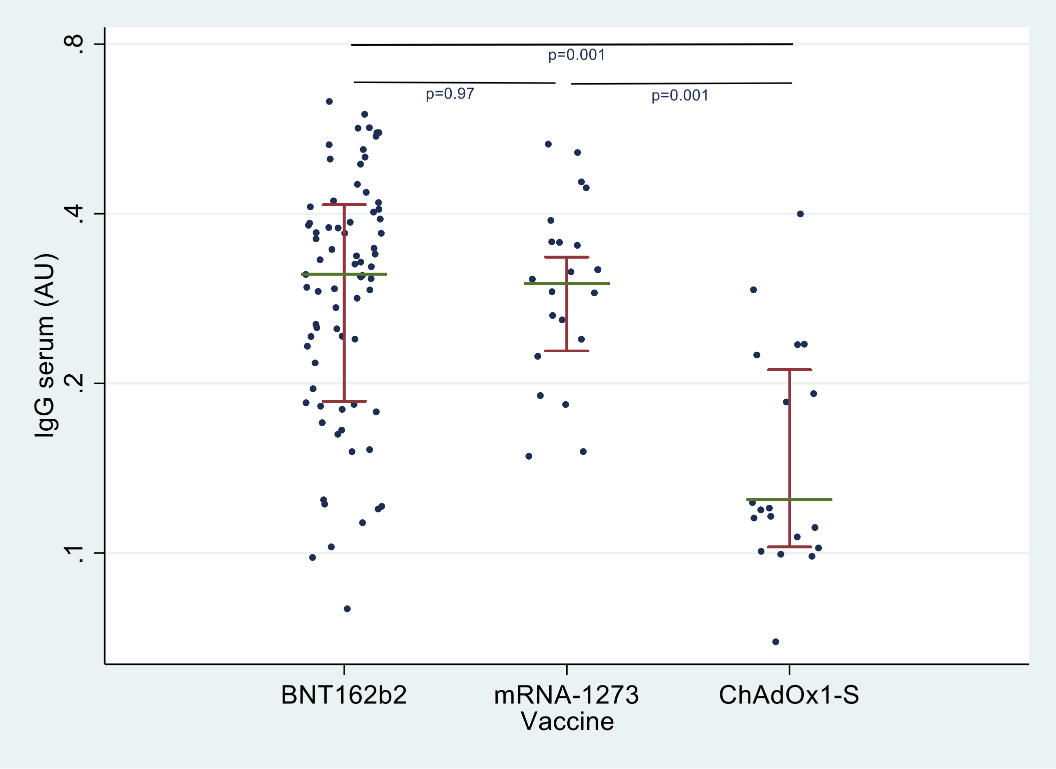

Supplementary Figure S1. Levels of IgG and IgA in serum and breast milk, according to the type of vaccine received.

B) IgA serum

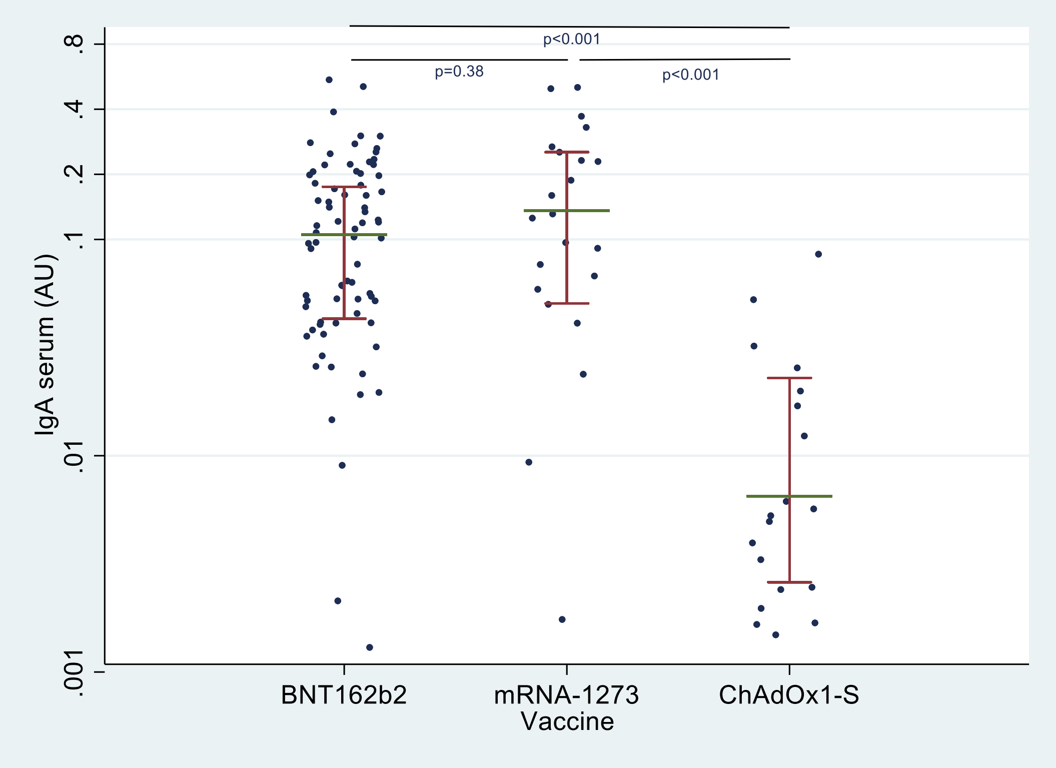

Supplementary Figure S1. Levels of IgG and IgA in serum and breast milk, according to the type of vaccine received.

C) IgG breast milk

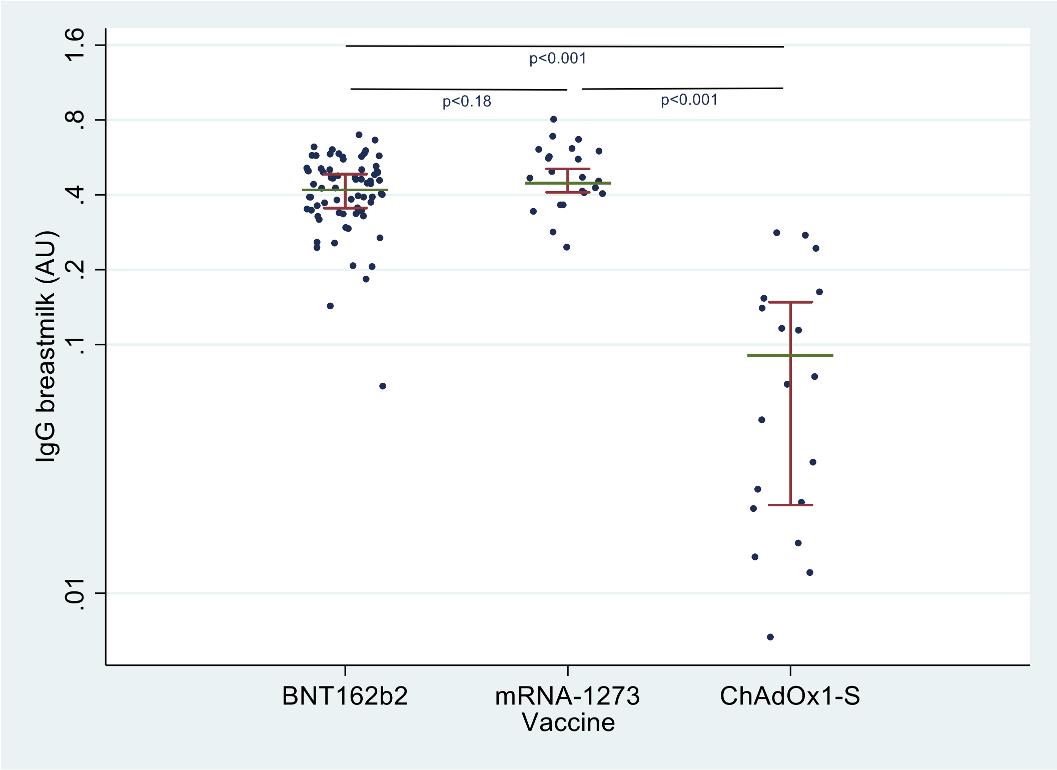

Supplementary Figure S1. Levels of IgG and IgA in serum and breast milk, according to the type of vaccine received.

D) IgA breast milk

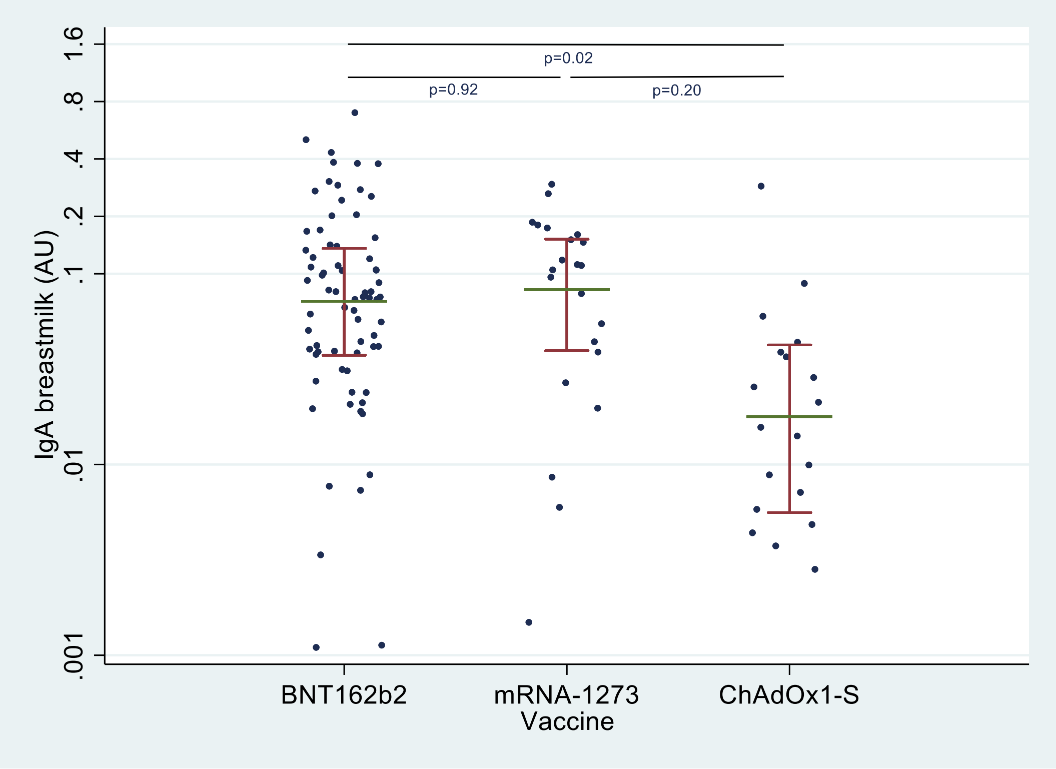

Supplementary Figure S2. Linear trend of the relationship between levels of IgG and IgA in serum and breast milk, according to the type of vaccine received.

A) Serum IgG and IgA

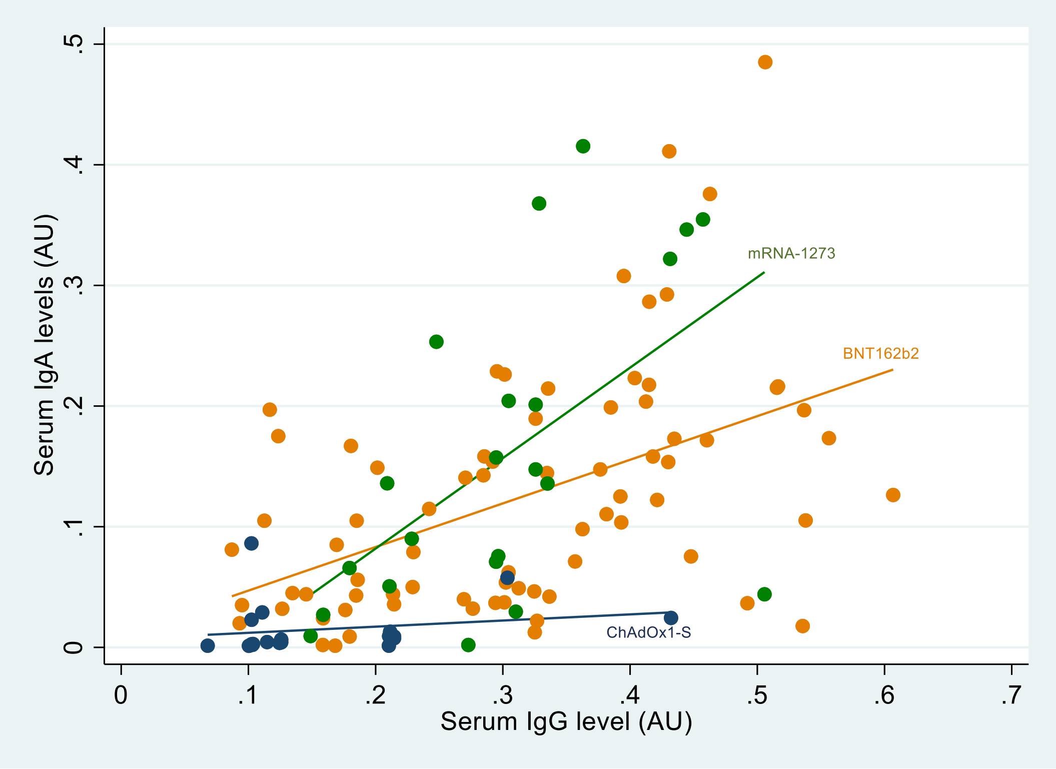

Supplementary Figure S2. Linear trend of the relationship between levels of IgG and IgA in serum and breast milk, according to the type of vaccine received.

B) Serum IgG and breastmilk IgA

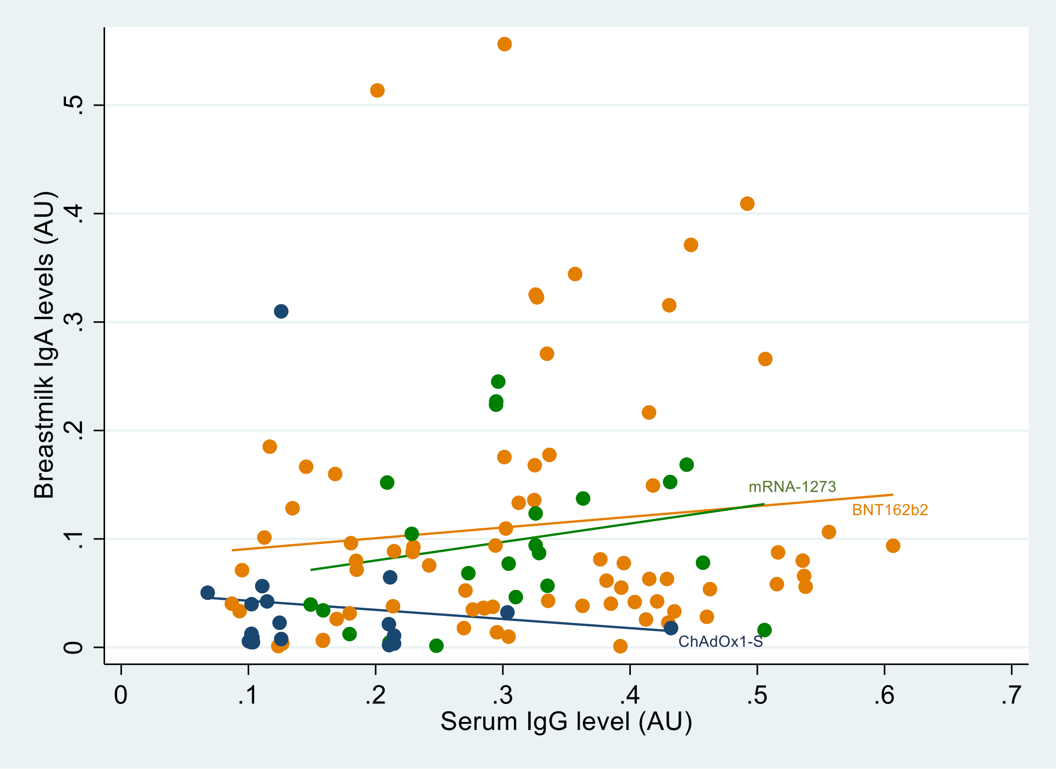

Supplementary Figure S2. Linear trend of the relationship between levels of IgG and IgA in serum and breast milk, according to the type of vaccine received.

C) Serum IgG and breastmilk IgG

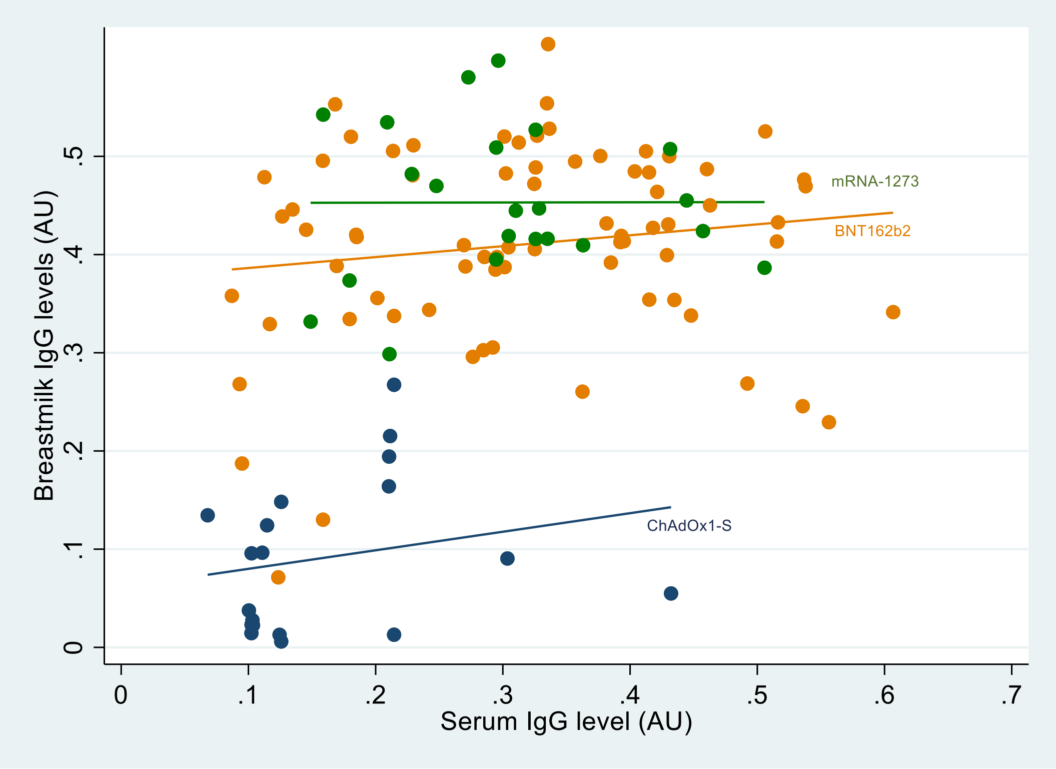

Supplementary Figure S2. Linear trend of the relationship between levels of IgG and IgA in serum and breast milk, according to the type of vaccine received.

D) Serum IgA and breastmilk IgA

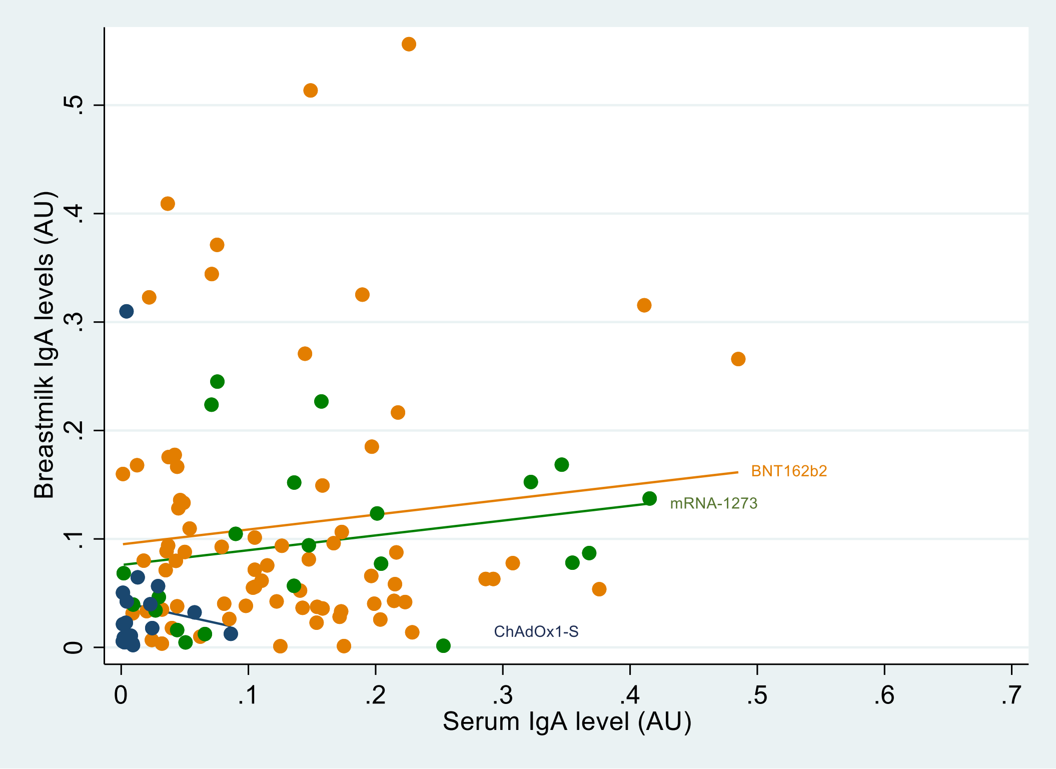

Supplement: Supplementary file 1 [file ijerph-18-08831-s001.zip › ijerph-1309372-SI.pdf]
